# Comparison of propofol‐based sedation and midazolam sedation in pediatric bidirectional endoscopy conducted by pediatric gastroenterologists

**DOI:** 10.1002/deo2.391

**Published:** 2024-06-14

**Authors:** Takahiro Kudo, Satomi Nishimoto, Ichitaro Horiuchi, Shingo Kurasawa, Satoshi Ukai, Akira Horiuchi

**Affiliations:** ^1^ Digestive Disease Center Showa Inan General Hospital Nagano Japan; ^2^ Department of Pediatrics Juntendo University Faculty of Medicine Tokyo Japan; ^3^ Department of Pediatrics Osaka Medical and Pharmaceutical University Osaka Japan; ^4^ Department of Gastroenterology Shinshu University Hospital Nagano Japan

**Keywords:** bidirectional endoscopy, children, midazolam, patient time requirement, propofol, sedation

## Abstract

**Objectives:**

The effectiveness and safety of propofol‐based sedation and midazolam sedation in pediatric bidirectional endoscopy were compared.

**Methods:**

We retrospectively analyzed the cases of pediatric patients (≤15 years old) who had undergone bidirectional endoscopy, esophagogastroduodenoscopy, and colonoscopy by pediatric gastroenterologists. Demographic data, indications, sedatives/dosages, clinical outcomes, endoscopic findings, adverse events, and total patient time requirements (total time in which patients stay in our hospital) were compared in the two sedation groups.

**Results:**

Ninety‐one children (51 boys, 40 girls, mean age 13 years, range 9–15) treated at our hospital were enrolled. Propofol alone or in combination with midazolam and/or pentazocine was administered to 51 patients (propofol‐based sedation group). Midazolam alone or in combination with pentazocine was administered to the other 40 patients (midazolam sedation group). In the propofol group, the following mean doses were used: propofol, 96 mg (range 40–145 mg); midazolam, 4.9 mg (range 3–5 mg); and pentazocine, 7.5 mg. In the midazolam group, the mean doses of midazolam and pentazocine were 6.2 mg (range 4–10 mg) and 15 mg, respectively. All procedures were successfully completed by pediatric gastroenterologists. The total procedure times and endoscopic findings were similar in the two groups, but the median patient time requirement in the propofol group was significantly shorter versus the midazolam group (7.3 h vs. 8.4 h, *p* < 0.001). No adverse events occurred in either group.

**Conclusions:**

Propofol‐based sedation in pediatric bidirectional endoscopy was safely and effectively performed by pediatric gastroenterologists, and its patient time requirement was shorter than that for midazolam sedation.

## INTRODUCTION

The effectiveness of same‐day bidirectional endoscopy, that is, the execution of both esophagogastroduodenoscopy (EGD) and colonoscopy procedures, has been reported for adult patients.^1^, ^2^, ^3^ The benefits of same‐day bidirectional endoscopy include a shorter hospital stay, reduced medical costs, and a reduction of the total sedation dose. Regardless of which comes first, EGD or colonoscopy, same‐day bidirectional endoscopy may also contribute to a quicker diagnosis of gastrointestinal diseases such as gastrointestinal bleeding, inflammatory bowel disease, and eosinophilic gastroenteritis, and it arguably improves the quality of health care.^4^

Propofol is a short‐acting sedative drug with a short recovery time.^5^ We have routinely used propofol for sedation in accordance with the revised (2nd edition) Japan Gastroenterological Endoscopy Society and the Japanese Society of Anesthesiologists ‘Guidelines for sedation in gastroenterological endoscopy’ to address on‐site clinical questions and any issues concerning safe examinations and treatment with the use of sedated endoscopy.^6^ After those guidelines' approval of the use of propofol sedation by gastroenterologists, the use of propofol sedation has been steadily increasing in gastrointestinal endoscopy for adults in Japan.

We have demonstrated that our hospital has safely established a policy of routine discharge of healthy adults to normal activity at 1 h after they have undergone sedated EGD or colonoscopy using propofol sedation.^7^, ^8^, ^9^, ^10^ We speculate that this policy has contributed to a decrease in the patient time requirement for this dual procedure. The ‘patient time requirement’ includes all phases of the patient's involvement: in‐hospital bowel preparation, waiting and preparing, the procedure, and recovery.

Propofol has also been successfully used in endoscopic procedures for children.^11^, ^12^, ^13^, ^14^ When propofol sedation is administered to pediatric patients by trained non‐anesthesiologists, it provides an excellent level of procedural success, fast recovery, and maximal patient comfort without an increase in adverse events.^14^ However, the benzodiazepine midazolam is still widely used for sedation. We have observed that propofol‐based sedation for EGD as administered by pediatric gastroenterologists greatly improved the visualization of the Z‐line in the induction of esophageal peristalsis in children and adolescents.^15^ We hypothesized that the optimal sedation practices could reduce the time requirement for these patients, but no data are available concerning the effectiveness and safety of propofol‐based sedation compared to midazolam sedation in pediatric bidirectional endoscopy. The present study was a retrospective analysis and comparison of the effectiveness and safety of propofol‐based sedation versus midazolam sedation for pediatric bidirectional endoscopy performed by pediatric gastroenterologists.

## METHODS

### Study design

This was a retrospective, cross‐sectional analysis of endoscopy results from a single center (Showa Inan General Hospital). The hospital's Institutional Review Board approved the retrospective chart review study protocol (No. 2022‐05, December 15, 2022). All subjects or their guardians had given written informed consent for the original procedures. The study was conducted in accordance with the Declaration of Helsinki. All authors had access to the study data and reviewed and approved the final manuscript.

### Patients

The cases of children between the ages of 9 and 15 who had undergone diagnostic bidirectional endoscopy (EGD + colonoscopy) with either propofol‐based or midazolam sedation at the Digestive Disease Center, Showa Inan General Hospital between January 2005 and December 2022 were analyzed. All patients enrolled in this study were limited to patients with the American Society of Anesthesiologists physical status classification I or II. Propofol alone or in combination with midazolam and/or pentazocine was defined as a propofol‐based sedation group. Midazolam alone or in combination with pentazocine was defined as the midazolam sedation group. The indications for bidirectional endoscopy were gastrointestinal symptoms (recurrent abdominal pain, vomiting, nausea, regurgitation, and dyspepsia), screening and surveillance for peptic ulcer, inflammatory bowel disease or eosinophilic gastroenteritis, gastrointestinal bleeding, and anemia. The cases of patients who had undergone a therapeutic endoscopy procedure including foreign body removal, hemostasis, or percutaneous endoscopic gastrostomy or endoscope‐guided duodenal tube placement were excluded. Repeat bidirectional endoscopies on already‐included subjects were included but counted as separate cases. Data regarding age, gender, weight, height, indications for bidirectional endoscopy, drugs used, endoscopic findings, and adverse events were obtained from the patients' electronic medical records.

### In‐hospital bowel preparation method

All of the patients were allowed regular meals the day before their procedure; no pretreatment was required the day before the colonoscopy.^16^ The regimen used at our hospital involved an initial intake of 5–10 mL of sodium picosulfate and/or 50 g of magnesium citrate (Horii Pharmaceutical Co.) dissolved in 0.2 L of water and the subsequent intake of 0.2 L of PEG‐Asc +0.2 L of water repeated 6–7 times with 15 min between administrations.

### Procedures

All bidirectional endoscopies including sedation without tracheal intubation were performed by experienced pediatric gastroenterologists.^10^, ^15^ The sedation drugs and dosages and the sequence of the EGD and colonoscopy procedures were determined at the pediatric gastroenterologist's discretion. All sedation drugs including propofol were administered by a nurse under the supervision of the pediatric gastroenterologist. Adequate sedation was considered to have been achieved when the patient passed through the following sequence: eyes closing, one or two yawns, and cessation of body movements. The target level of sedation was moderate sedation with patients being able to respond purposefully to verbal or tactile stimulation. When its target level was not obtained or the patients were undersedated, an additional injection of 20 mg of propofol or 1 mg of midazolam was given.

EGDs or ileocolonoscopies were performed using either a small‐caliber or standard upper endoscope (GIF‐XP 240, XP260, or H290; Olympus or PCF‐Q260AZI, or PCF‐H290ZI; Olympus) with an endoscopic video information system (CV 240, CV260, or CV290; Olympus). The endoscopic images were recorded by both computer images and a text reporting system. Cardiovascular monitoring and continuous oximeter measurement were conducted for all patients. Respiratory or cardiovascular complications were recorded by the attending nurse at the time of the procedure. Supplemental oxygen was given to all patients to maintain oxygen desaturation levels above 93% during the endoscopic procedures.

### Evaluated outcomes

Demographic data, indications, anesthetic drugs and dosages, the endoscopic sequence, total procedure times, adverse events, endoscopic findings, and total patient time requirements (the total time in which patients stay in our hospital) were compared between the groups of patients who received propofol‐based sedation or midazolam sedation. The total procedure time was defined as the time from the initial insertion of the endoscope to its withdrawal. Recovery time was thought of as the time from the removal of the endoscope until the patient became fully alert.

### Statistical analyses

Differences in the categorical variables were analyzed by the χ^2^‐test or, when appropriate, Fisher's exact test. The continuous data are expressed as the mean ± standard deviation and were compared by Student's *t*‐test. Non‐parametric data were analyzed by Wilcoxon's rank sum test. Statistical significance was taken as a two‐sided p‐value <0.05. The statistical analyses were performed using GraphPad Prism ver. 10.1.1 (GraphPad Software Inc.).

## RESULTS

Table 1 summarizes the baseline characteristics and indications in pediatric patients with bidirectional endoscopy in the propofol‐based sedation and midazolam sedation groups. The cases of a total of 91 children (51 boys, 40 girls; mean age 13 years, range 9–15 years) were analyzed. The demographic characteristics and indications were similar in the two groups. Propofol alone or in combination with midazolam and/or pentazocine was administered to 51 patients (the propofol‐based sedation group) while midazolam alone or in combination with pentazocine was administered to the other 40 patients (midazolam sedation group; Table 2). In the propofol‐based sedation group, the following mean doses were used: propofol, 96 mg (range 40–165 mg); midazolam, 4.9 mg (range 3–5 mg); and pentazocine, 7.5 mg. In the midazolam sedation group, the mean doses of midazolam and pentazocine were 6.2 mg (range 4–10 mg) and 15 mg, respectively.

**TABLE 1:** Baseline characteristics and indications in pediatric patients with bidirectional endoscopy with either propofol‐based sedation or midazolam sedation.

	Propofol‐based	Midazolam	*p‐*value
Patients, *n*	51	40	
Age, years^a^ mean (SD)	12.7 (1.8)	13.1 (1.5)	0.54
Male^b^	30	21	0.67
Body height, cm^a^ mean	158	154	0.29
Body weight, kg^a^ mean	47.1	45.1	0.28
Indications^b^			0.33
GI symptoms	39	29	
Screening	4	3	
Surveillance	2	6	
GI bleeding	5	2	
Anemia	1	0	

Abbreviation: GI, gastrointestinal.

^a^ Differences between the propofol‐based and midazolam sedation groups were compared by Student's t‐test for continuous variables.

^b^ Differences between the propofol and midazolam groups compared by χ^2^‐test or, when appropriate, Fisher's exact test for categorical data.

**TABLE 2:** Sedation drugs and doses used in pediatric patients with bidirectional endoscopy with either propofol‐based sedation or midazolam sedation.

	Propofol‐based *n* = 51	Midazolam *n* = 40	*p‐*value
Sedation drugs and doses used:			
Propofol alone, 40–145 mg	8		
Propofol + pentazocine 7.5 mg	2		
Propofol + midazolam 3–5 mg	29		
Propofol + midazolam 4 mg + pentazocine 7.5 mg	12		
Midazolam, 4–10 mg		5	
Midazolam, 4–10 mg + pentazocine, 15 mg		35	
Mean propofol, mg	96		
Mean midazolam, mg	4.9	6.2	<0.001
Mean pentazocine, mg	7.5	15	<0.001

*Note*: Differences between the propofol‐based and midazolam sedation groups were compared by Student's t‐test for continuous variables.

Table 3 shows the clinical outcomes in the propofol‐based sedation and midazolam sedation groups. Although the endoscopic sequence differed significantly between the two groups (p<0.001), the two groups showed similar mean EGD procedure times, mean colonoscopy times, and mean total procedure times. No adverse events such as cardiopulmonary events, perforation, nausea, or vomiting occurred in either group (Table 3).

**TABLE 3:** Clinical outcomes in pediatric patients with bidirectional endoscopy with either propofol‐based sedation or midazolam sedation.

	Propofol‐based *n* = 51	Midazolam *n* = 40	*p‐*value
Endoscopic sequence^b^			
EGD to colonoscopy	19	30	<0.001
Colonoscopy to EGD	32	10	
Mean EGD procedure time, min^a^	9	8	0.34
Mean colonoscopy procedure time, min^a^17	15	0.54	
Cecal intubation rate, %^b^	94.1	97.5	0.50
Mean cecal intubation time, min^a^	9	6	0.44
Mean withdrawal time, min^a^	8	8	0.88
Mean total procedure time, min^a^	27	23	0.65
Biopsy taken^b^	48 (94%)	40 (100%)	0.25
Mask ventilation required	0 (0%)	0 (0%)	
Heart rate <50 beats/min	0 (0%)	0 (0%)	
Perforation	0 (0%)	0 (0%)	
Nausea and vomiting	0 (0%)	0 (0%)	

Abbreviation: EGD, esophagogastroduodenoscopy.

^a^ Differences between the propofol and midazolam groups were compared by Student's t‐test for continuous variables.

^b^ Differences between the propofol and midazolam groups were compared by χ2‐test or, when appropriate, Fisher's exact test for categorical data.

Table 4 lists the endoscopic findings in the two sedation groups. There were no significant differences in EGD or colonoscopy endoscopic findings between the groups. Table 5 provides the patient time requirements. With regard to the median durations of in‐hospital bowel preparation, waiting and preparing, and the sum of the two endoscopy procedures themselves, there were no significant differences between the two groups. However, the median total patient time requirement, including these three components plus the recovery time, was significantly shorter in the propofol‐based group than in the midazolam group (7.3 h vs. 8.4 h, *p* < 0.001).

**TABLE 4:** Endoscopic findings in pediatric patients with bidirectional endoscopy with either propofol‐based sedation or midazolam sedation.

	Propofol‐based *n* = 51	Midazolam *n* = 40	*p‐*value
EGD			0.29
Normal	27	17	
Reflux esophagitis	9	15	
Chronic gastritis	6	1	
Duodenal ulcer & duodenitis	4	5	
Crohn's disease	2	2	
Eosinophilic gastroenteritis	1	2	
Others	6	6	
Colonoscopy			0.43
Normal	30	32	
Ulcerative colitis	6	2	
Crohn's disease	2	3	
Proctitis	2	1	
Nonspecific colitis	3	1	
Others	4	1	

*Note*: Differences between the propofol and midazolam groups were compared by χ2‐test or, when appropriate, Fisher's exact test for categorical data.

Abbreviation: EGD, esophagogastroduodenoscopy.

**TABLE 5:** Patient time requirements in pediatric patients with bidirectional endoscopy with either propofol‐based sedation or midazolam sedation.

	Median no. of hours (range)		
	Propofol‐based *n* = 51	Midazolam *n* = 40	*p‐*value
In‐hospital bowel preparation	3.2 (3–5)	3.5 (3–5)	0.57
Waiting and preparing	1.5 (1–2)	1.4 (1–2)	0.42
Total procedure time	0.5	0.5	0.73
Recovery time	2.1	3.0	<0.001
Total patient time requirements	7.3 (6–9)	8.4 (7–10)	<0.001

*Note*: The total procedure time was defined as the time from the initial insertion of the endoscope to its withdrawal. Recovery time was thought of as the time from the removal of the endoscope until the patient became fully alert. Total patient time requirements: the total time that the patients stayed in our hospital for bidirectional endoscopy. Differences between the propofol and midazolam groups were compared by Wilcoxon's rank sum test.

## DISCUSSION

All of the procedures were successfully completed by pediatric gastroenterologists and all patients were discharged home, irrespective of the sedation method. The patient time requirements in clinical practice are always critical. Our present findings demonstrated that the patient time requirements in bidirectional endoscopy depended on the sedation agents used. As shown in Table 2, the mean doses of midazolam and pentazocine used in the propofol‐based sedation group were much lower than those of the midazolam group (4.9 and 7.5 mg in the propofol group vs. 6.2 and 15 mg in the midazolam group).

Because of the obstacles encountered when attempting to measure the patients' ‘recovery time’ per se from the existing medical records, we simply evaluated the total patient time requirement, essentially the time spent in the hospital, in this study. The results of our analyses demonstrated that the median total patient time requirement in the propofol‐based sedation group was significantly shorter than that of the midazolam sedation group (7.3 h vs. 8.4 h, p<0.001), although no significant between‐group differences were detected for three of the four components of the total patient time requirement: that is, the in‐hospital bowel preparation time, the waiting and preparing time, and the total procedure time. The most likely reason for the significantly shorter total patient time requirement in the propofol‐based sedation group is that the use of propofol allowed a decrease in the dose of midazolam and/or pentazocine, resulting in a decrease in the recovery time required. Midazolam (a sedative) and pentazocine (an anesthetic with sedation effects) are both longer‐acting than propofol and are generally administered together for a synergistic effect; however, the synergistic effect of these drugs also delays recovery, necessitating a longer hospital stay.

The sedation drugs and dosages used and the sequence of EGD and colonoscopy in the present series of pediatric patients were determined at the pediatric gastroenterologist's discretion. As various sedation protocols were included in this study, they were classified as either propofol‐based or midazolam sedation. The reason that propofol was sometimes used in addition to midazolam in the propofol‐based sedation group seems to be that pediatric gastroenterologists expected a synergistic sedation action of propofol over midazolam alone for bidirectional endoscopy. We thus compared the effectiveness and safety of the sedation protocols in this study based on the presence or absence of propofol, that is, propofol‐based sedation or midazolam sedation.

Propofol is contraindicated for sedation during ventilation in intensive care because of the high risk of propofol infusion syndrome in children. So, the use of excessive doses of propofol must be avoided, even if it is used for gastrointestinal endoscopic sedation. Moreover, we must recognize that the manufacturers of propofol restrict its use solely to personnel trained in general anesthesia. The dose of propofol and midazolam are usually expressed per kg body weight. However, in our previous studies propofol or midazolam was administered by a nurse via a bolus injection following an age‐adjusted standard protocol.^7^, ^8^, ^9^, ^10^ As there were no significant differences in body height or body weight between the present two sedation groups (Table 1), the comparison of the mean doses of sedation drugs used in these patients may be accepted.

It has been reported that same‐day bidirectional endoscopy with the administration of midazolam and pethidine significantly increased the incidence of both hypoxia and post‐endoscopic nausea compared to propofol administration.^17^ Nausea and vomiting might be the result of stimulation of the medullary chemoreceptor trigger zone by pethidine.^18^ Our present analyses did not detect nausea or vomiting as adverse events in any of the enrolled children, regardless of the sedation used. However, it was possible that very mild nausea could have potentially occurred, and it may have delayed recovery as greater doses of midazolam and pentazocine were used in the midazolam sedation group than the propofol‐based sedation group.

In addition, the financial balance of sedation depends on the drugs used.^19^ Even if sedation is safe, it requires an extra nurse and a monitoring‐trained team, resulting in higher costs when the patient's recovery time is long. In the patient cases examined in our present investigation, the social costs of conventional sedation with the combination of midazolam and pentazocine (e.g., increased recovery time, inability to go home or return to school, and the need for a companion) may have been higher than those of propofol‐based sedation.

Based on a systematic review and meta‐analysis, the optimal sequence of same‐day bidirectional endoscopy is EGD followed by colonoscopy.^4^ In the present study, the sequences of bidirectional endoscopy were significantly different between the two groups (Table 3). It is unknown whether the sequence of bidirectional endoscopy was associated with shorter recovery time in this study.

In the United States, bowel preparation is done by the patient at home; the typical policy is ‘no food for 12 h, plus the use of magnesium citrate.’ In Japan, bowel preparation is commonly an in‐hospital experience. All of the present patients were allowed regular meals the day before their procedure; no pretreatment was required the day before the colonoscopy. As all 91 of the children underwent bowel preparation in our hospital, we described our bowel preparation method as ‘in‐hospital bowel preparation.’ We suspect that this bowel preparation method itself may not be associated with the effectiveness and safety of these two sedation methods.

Our study has some limitations. It was retrospective and was conducted at an endoscopy unit in a single community hospital in Japan where the nurses and technicians were highly trained. The number of pediatric patients enrolled was relatively small (*n* = 91). In addition, it might be not possible to eliminate the influence of the patients' backgrounds or other potential confounding factors. A follow‐up study should be performed prospectively to confirm and clarify the characteristics of adverse events after bidirectional endoscopy.

In conclusion, propofol‐based sedation as well as midazolam sedation was safely performed for pediatric bidirectional endoscopies by pediatric gastroenterologists. The benefits of same‐day bidirectional endoscopy in children may be enhanced by propofol‐based sedation rather than midazolam sedation because of the former's shorter patient time requirement.

## CONFLICT OF INTEREST STATEMENT

None.

## Data Availability

The data underlying this article will be shared on reasonable request to the corresponding author.
